# Steroid-refractory ulcerative colitis treated with corticosteroids, metronidazole and vancomycin: a case report

**DOI:** 10.1186/1471-230X-5-3

**Published:** 2005-01-24

**Authors:** Jonathan Miner, M Monem Gillan, Philip Alex, Michael Centola

**Affiliations:** 1 University of Oklahoma Health Sciences Center, Oklahoma City, OK, 73104 USA; 2 Oklahoma Medical Research Foundation, Oklahoma City, OK, 73104 USA

## Abstract

**Background:**

Increasing evidence elucidating the pathogenic mechanisms of ulcerative colitis (UC) has accumulated and the disease is widely assumed to be the consequence of genetic susceptibility and an abnormal immune response to commensal bacteria. However evidence regarding an infectious etiology in UC remains elusive.

**Case presentation:**

We report a provocative case of UC with profound rheumatologic involvement directly preceded by *Clostridium difficile *infection and accompanying fever, vomiting, bloody diarrhea, and arthritis. Colonic biopsy revealed a histopathology suggestive of UC. Antibiotic treatment eliminated detectable levels of enteric pathogens but did not abate symptoms. Resolution of symptoms was procurable with oral prednisone, but tapering of corticosteroids was only achievable in combination therapy with vancomycin and metronidazole.

**Conclusions:**

An infectious pathogen may have both precipitated and exacerbated autoimmune disease attributes in UC, symptoms of which could be resolved only with a combination of corticosteroids, vancomycin and metronidazole. This may warrant the need for more perceptive scrutiny of *C. difficile *and the like in patients with UC.

## Background

*Clostridium difficile *infection may be a compounding factor in ulcerative colitis (UC) with data suggestive of a role in disease exacerbation or initiation by the organism in a subset of patients [1]. Adjunctive antibiotic therapy, either broad-spectrum or gram positive-specific, has shown limited efficacy in UC [2,3]. However, sporadic reports in which significant therapeutic potential was achieved [4-6] suggest that a distinct subclass of patients with an infectious-associated phenotype may exist. Although there have been an increasing number of case reports on ulcerative colitis complicated by cytomegalovirus infection [7-9], there are few with conclusive evidence of infection with *Clostridium difficile*. Herein, we report a case of steroid-resistant UC with profound rheumatologic involvement immediately preceded by *C. difficile *infection in which remission of UC symptoms was twice achieved in response to adjunct metronidazole and vancomycin therapy. The patient remained symptom-free without supportive therapy for a four-year period.

## Case presentation

A 32-year-old white male presented with nausea, vomiting, crampy lower abdominal pain and diarrhea. His initial lab values were hemoglobin 15.6 g/dl (14–18 g/dl), hematocrit 45.8% (40–54%), sodium 131 mEq/l (135–146 mEq/l), potassium 3.9 mEq/l (3.5–5.5 mEq/l), bicarbonate 24 mEq/l (22–24 mEq/l), chloride 91 mEq/l (95–112 mEq/l), BUN 37 mg/dl (7–25 mg/dl), serum creatinine 1.6 mg/dl (0.7–1.4 mg/dl), and normal liver function tests. He was treated with intravenous fluids, Ciprofloxacin and anti-emetics, to which the diarrhea resolved in 2–3 days. After one month he had recurrent watery diarrhea accompanied with rectal pain, bleeding and vomiting. He also developed fever of 101.6 F.

The physical exam was remarkable for decreased bowel sounds and moderate lower abdominal tenderness. There were no masses or organomegalies palpable. The rectal exam was remarkable for tenderness and heme-positive mucus. The remainder of the physical exam was uneventful. Stool samples from the initial visit were positive for *Clostridium difficile *cytotoxin, and depicted many WBCs (neutrophils 99% and eosinophils 1%), suggesting an infectious etiology. Stool sample was negative for giardia lamblia, salmonella, shigella, campylobacter, aeromonas and plesiomonas. There was no predominance of staphylococcus, yeast, or pseudomonas.

The patient was initially started on Ciprofloxacin (500 mg bid.) for 3 days without symptom resolution. Metronidazole was added (250 mg tid.), but intolerance of the patient to metronidazole with exacerbations of nausea prompted the replacement to Vancomycin (250 mg qid) in conjunction with Ciprofloxacin for 7 days. During this initial treatment period, the patient characteristically developed migratory polyarticular joint pain and swelling involving the shoulders, elbows, fingers, hips and knees. Lack of response to antibiotics suggested an autoimmune disease as a contributing factor.

**Figure 1:**
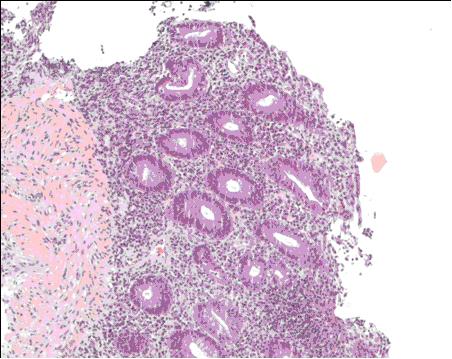
Colonic mucosal inflammation in ulcerative colitis with loss of goblet cells and neutrophilic infiltrate (magnification ×100)

At this time a sigmoidoscopy and pinch biopsy revealed diffuse acute and chronic inflammation with cryptitis, mucin depletion, and glandular foreshortening and branching suggestive of UC (Fig. 1). The patient was treated with Asacol (400 mg bid.) in conjunction with metronidazole (250 mg qid). After 7 days of combined therapy with continued weight loss and no improvement, prednisone (60 mg qid.) was added, resulting in cessation of GI symptoms and moderate improvement in joint pain and swelling. After 7 days of continued improvement, the antibiotic and Asacol therapy were stopped. The patient was maintained on high dose steroids for 14 days. However, fever, gastrointestinal, and joint symptoms recurred upon tapering of prednisone to 20 mg a day. When symptoms recurred, a stool culture was negative for infectious agents including *Clostridium difficile *antigen, confirming an autoimmune component to disease pathology in this patient. Prednisone was increased to 30 mg per day at which point gastrointestinal symptoms resolved but joint swelling and pain continued. Over the course of 3 months prednisone tapering was attempted three additional times, with each resulting in symptom recurrence.

After 3 months of treatment, the patient experienced an acute dental abscess and was prescribed a combination vancomycin (250 mg tid) and metronidazole (250 mg tid). During this period, the patient was able to gradually reduce prednisone dosage without symptom recurrence. Antibiotics were continued for 30 days during which time prednisone was tapered completely. Symptoms did not recur and the patient remained symptom-free without further therapeutic intervention for 4.5 years.

After this prolonged period of remission of UC symptoms, a second episode similar to the first occurred. The patient once again presented with bloody diarrhea, nausea, vomiting, and joint pain. Stool cultures were *Clostridium difficile *positive by culture and cytotoxin. Once again the patient was treated with 60 mg daily of prednisone and metronidazole (250 mg tid). Symptoms did not resolve, and vancomycin (125 mg tid) was added, resulting in a significant improvement in symptoms. After 2 weeks of combined therapy metronidazole treatment was stopped and symptoms recurred within a week. Metronidazole was once again added, leading to resolution of symptoms. Combined antibiotic and steroid therapy continued for 2 months during which time prednisone was tapered completely and a second remission of UC symptoms was induced.

## Discussion

The patient was remarkable in several regards. The onset of UC and spondyloarthropathy coincided with *C. difficile *infection. While various therapeutic regimens of prednisone, vancomycin and metronidazole preceded a negative *C. difficile *toxin test, remission of UC symptoms could only be induced by the combination of these three medications. *C. difficile *toxin tests have shown varying efficacy in diagnosing pseudomembranous colitis. It has been demonstrated that toxin-negative patients with *C. difficile *positive stool culture may still have symptomatic pseudomembranous colitis [10]. Lynch et al showed that 46% of stool specimens from patients test negative by cytotoxin but positive by culture [11]. Likewise, others have shown that some cases of *C. difficile *infection cannot be detected by the cytotoxin assay and suggested that the organism has the ability to vary toxin production [12-16]. The bacterium also tends to persist as an antibiotic-resistant vegetative spore, which explains the high frequency at which the symptoms recur following treatment.

Since toxin-negative patients have presented with *C. difficile *related diarrhea, it is reasonable to assume that the organism may exist undetectably in a symptomatic patient, but especially in a patient with inflammatory bowel disease (IBD). The elusiveness of the bacterium in diagnosis of *C. difficile *infected IBD patients was confirmed in 2001 by Markowitz et al. They demonstrated that single-toxin assays failed to identify *C. difficile *infection in approximately 40% of pediatric IBD patients and in a case report where a toxin A positive, toxin B negative variant was detected [14,17]. It has also been shown that IBD patients have a higher incidence of *C. difficile *infection than in healthy controls [18]. When considered together with the case reported here, this information leads us to the hypothesis that a small subclass of UC pathology may actually have an infectious etiology resulting from chronic *C. difficile *infection.

IBD has been shown to be more prevalent in industrialized nations, where antibiotic treatment has its longest history [19]. The increase in IBD prevalence parallels rising antibiotic use over the last 50 years [20]. If an undetected antibiotic-resistant enteric pathogen is responsible for inducing or exacerbating IBD, then widespread antibiotic use may have contributed to the increased prevalence of IBD in these industrialized nations.

This case is especially interesting considering *H. pylori *and its recently discovered tendency to adhere to glycoconjugates expressed in inflamed gastric mucosa [21]. In light of this new information, it is tempting to speculate that *C. difficile *could similarly bind to inflamed colonic tissue in this subclass of UC patients. Further research in this area could yield valuable new data.

The potential utility of combined therapy is further suggested by the fact that antimicrobial resistance among C. difficile strains to metronidazole and with intermediate resistance to vancomycin is emerging in countries like Hong Kong (where one of 100 C. difficile isolates was resistant to metronidazole) and Spain (where 9% of 469 clinically significant C. difficile isolates were resistant to metronidazole, particularly in isolates recovered from HIV-positive patients and few patients also had intermediate resistance to vancomycin) [22-24] It is interesting to note that no resistance was found to metronidazole or vancomycin among C. difficile strains from isolates in patients from UK, Germany, Brazil, Poland or Kuwait [25-29]. The Public Health Laboratory Service (PHLS) Anaerobe Reference Unit (ARU) has also not been successful in detection of metronidazole resistance in any of their over 1000 C. difficile isolates tested [24]. The impact of drug resistance should be considered if long-term treatment is utilized in patients. However, a complicating factor in this context is that for metronidazole and vancomycin, minimum inhibitory concentration susceptibility breakpoints are usually set for isolates causing systemic infections that are based upon antimicrobial levels in blood (serum) and not on the levels in the intraluminal area, where higher drug concentrations can be achieved [30].

## Conclusions

Recurrent relapses could only be suppressed in this patient by the combination therapy of corticosteroids, metronidazole and vancomycin. Our observations suggest that this combination therapy may be effective to confront and induce remissions of UC symptoms in patients with *C. difficile *toxin-positive refractory autoimmune symptomatologic UC as opposed to the use of a single agent.

An opportunistic *C. difficile *infection commonly results from the use of broad-spectrum antibiotics like quinolones. The frequent use of these antibiotics in treating IBD suggests that these patients could develop *C. difficile *infection during treatment, thereby exacerbating symptoms and preventing remission of UC symptoms. It may therefore be practical to probe for *C. difficile *infections more meticulously in patients with IBD.

## Competing interests

The author(s) declare they have no competing interests.

## Authors' contributions

All authors contributed equally to this work. All authors read and approved the final manuscript.

## Pre-publication history

The pre-publication history for this paper can be accessed here:
